# Impact of Vitamin D Status and Supplementation on Brain-Derived Neurotrophic Factor and Mood–Cognitive Outcomes: A Structured Narrative Review

**DOI:** 10.3390/nu17162655

**Published:** 2025-08-16

**Authors:** Aleksandra Skoczek-Rubińska, Angelika Cisek-Woźniak, Marta Molska, Martyna Heyser, Martyna Trocholepsza, Sebastian Pietrzak, Kinga Mruczyk

**Affiliations:** 1 Department of Dietetics, Faculty of Physical Culture in Gorzów Wielkopolski, Poznan University of Physical Education, 66-400 Gorzów Wielkopolski, Poland; a.cisek@awf-gorzow.edu.pl (A.C.-W.); m.molska@awf-gorzow.edu.pl (M.M.); martynahey@vp.pl (M.H.); martyna.trocholepsza@o2.pl (M.T.); k.mruczyk@awf-gorzow.edu.pl (K.M.); 2 Sebastian Pietrzak Company, 66-400 Gorzów Wielkopolski, Poland; sebastian.pietrzak@interia.com

**Keywords:** vitamin D, brain-derived neurotrophic factor, cognition

## Abstract

Background/Objectives: Vitamin D deficiency is prevalent in higher-latitude regions and among older adults, and has been linked to depressive symptoms and cognitive decline, although the neurobiological link remains unclear. Brain-derived neurotrophic factor (BDNF) may be a key modulator and mediator of vitamin D-related neuroprotection. Methods: Selected databases (2009–2025) were searched for specific studies reporting vitamin D exposure, BDNF, and mood or cognitive outcomes. Risk of bias was appraised with RoB 2, Newcastle–Ottawa Scale or SYRCLE. Results: Thirteen studies were included. High-dose vitamin D improves mood primarily when levels are low. Supplementation of at least 2000 IU/day for 12 weeks reduced BDI scores by 1.7–7.6 points and increased BDNF levels by ~7%. Each 1 ng/mL increase in 25(OH)D levels decreased the likelihood of depressive symptoms, especially when BDNF levels were high. In animal studies vitamin D increases hippocampal BDNF and reverses stress-induced depressive behavioral deficits. Adequate vitamin D intake is associated with improved cognitive performance and a dose-dependent increase in BDNF. Each 10 ng/mL increase in 25(OH)D was associated with a 0.6-point increase in MMSE scores and a 15% increase in serum BDNF. Low vitamin D status in children may predict cognitive decline. Animal studies have shown that supplementation with 500–10,000 IU/kg for at least 3 weeks increased hippocampal BDNF and improved biochemical markers of aging. Conclusions: Vitamin D supplementation may support mood and cognition via BDNF modulation, especially in people with insufficient vitamin D levels (<30 ng/mL), but long-term, adequately powered studies with objective tools are required.

## 1. Introduction

Vitamin D is a secosteroid synthesized in the skin under ultraviolet-B irradiation and subsequently hydroxylated to 25-hydroxycholecalciferol [25(OH)D], the accepted biomarker of vitamin-D status. Hypovitaminosis D is a major global public health concern. A recent meta-analysis showed that 59.7% of individuals were vitamin D deficient (<20 ng/mL; <50 nmol/L), and among 6748 older adults, 27.5% were deficient (20–30 ng/mL; 50–75 nmol/L), with only 16.0% achieving a deficiency above 30 nmol or 75 nmol [1]. A comparative study showed that deficiency (<50 nmol/L) affected 70.3% of Chinese adults aged ≥ 65 years (severe deficiency < 30 nmol/L in 30.6%) compared to 17.4% in the USA (severe in 3.4%) [2]. In frail older hospitalized patients, deficiency (<20 ng/mL) was found in 87.4%, with only 38% achieving desirable levels (>30 ng/mL); the estimated pre-test probability of deficiency was 96% [3]. Certain neuropsychiatric groups are also at high risk: in hospitalized adolescents with psychiatric disorders, deficiency (<20 ng/mL) was present in 34% and insufficiency (20–30 ng/mL) in 38%, with psychotic features significantly more common in deficient patients [4]. According to global health analyses, vitamin D deficiency contributes substantially to disability-adjusted life years (DALYs) through its association with musculoskeletal, cardiovascular, and neuropsychiatric outcomes [5]. Vitamin D Receptor (VDR) is expressed in brain regions relevant to mood and cognition [6,7].

Brain-derived neurotrophic factor (BDNF) is the principal neurotrophin supporting neuronal survival and long-term synaptic potentiation; reduced BDNF has been linked to cognitive decline and mood disorders [8,9]. Chronic inflammation and oxidative stress—prevalent in depression and aging—suppress BDNF expression, establishing a cascade of inflammation → reduced BDNF → clinical symptoms [10].

Depression affects ~280 million individuals worldwide, with a prevalence of 5–20% in adults aged ≥65 years [11,12,13]. Mood deficits often co-occur with persistent cognitive impairment, increasing dementia risk [14,15,16]. Standard pharmacotherapy benefits only two-thirds of patients and carries adverse effects [17,18], prompting interest in nutritional approaches. Some observational and interventional studies consistently report that low 25(OH)D is associated with poorer cognitive performance [19,20,21,22,23] and greater depressive severity [24]. A meta-analysis from 2023 suggests that supplementation ≥ 2000 IU/day alleviates depressive symptoms and elevates circulating BDNF [25]. Vitamin D may exert its neurotrophic effects by activating intracellular pathways such as the cyclic adenosine monophosphate (cAMP) response element-binding protein (CREB) cascade, which regulates BDNF transcription and supports synaptic plasticity [26,27]. Preclinical data further suggest that vitamin D upregulates BDNF via CREB signaling [8,28,29], but the study by Yang et al. (2024) did not find a significant change in BDNF expression despite behavioral improvements, suggesting that other mechanisms may mediate the antidepressant effect [30].

The present article provides a narrative review, assessed with the Scale for the Assessment of Narrative Review Articles (SANRA), of clinical RCTs, observational and animal models studies published between 2009 and 2025 that quantified BDNF and examined the impact of vitamin D status or supplementation on BDNF and mood or cognitive outcomes. A systematic structure—search strategy, eligibility criteria, tabulated synthesis—is applied without a quantitative meta-analysis. Study quality was assessed with SANRA, RoB 2, NOS and the SYRCLE risk of bias tool in line with reporting guidance.

## 2. Materials and Methods

### 2.1. Study Design

This structured narrative review was conducted in accordance with the STROBE-Nut guidance [31] for nutritional epidemiology. Methodological quality was appraised with the Scale for the Assessment of Narrative Review Articles (SANRA) (Table 1) [32]. PRISMA guidelines were also used to prepare this review (Table 2) [33].

### 2.2. Eligibility Criteria

Eligible studies included randomized controlled trials (RCTs), observational studies (cross-sectional or cohort), and controlled animal studies published in English between 2009 and 2025. Human studies enrolled children or adults with or without mood or cognitive disorders and reported brain-derived neurotrophic factor (BDNF) and vitamin D intervention or serum 25(OH)D status as well as mood or cognition outcomes. In human interventional clinical trials, the vitamin D dosing regimen was recorded (e.g., 2000 IU/day or 50,000 IU/week), and in some cases baseline and postintervention 25(OH)D levels were collected when available.

Diagnostic evaluation of depressive symptoms was typically performed using validated self-report or clinician-administered instruments, such as the Beck Depression Inventory-II (BDI-II—A 21-item self-report questionnaire measuring the severity of depressive symptoms over the past two weeks) [34], the patient health questionnaire-9 (PHQ-9—a 9-item screening tool based on DSM-IV criteria for depression, commonly used to assess symptom severity and diagnostic likelihood) [35], or the Mini-International Neuropsychiatric Interview (MINI—A structured diagnostic interview used to assess major psychiatric disorders according to DSM and ICD criteria) [36]. Cognitive function in Quialheiro et al. (2023) was assessed using the mini-mental state examination (MMSE), a widely validated instrument adapted for the Brazilian population; the MMSE total score (range: 0–30 points) was analyzed as a continuous outcome in the path analysis models [19,37]. In Dewi et al. (2025) a children’s study on cognition was assessed using the Ages and Stages Questionnaire Third Edition (ASQ-3), a validated parent-report tool for children aged 1–66 months. It evaluates five developmental domains: communication, gross motor, fine motor, problem solving (used as a proxy for cognition), and personal-social. Lower scores in the problem-solving domain were interpreted as indicative of cognitive delay [20,38]. BDNF was typically measured using enzyme-linked immuno-sorbent assay (ELISA). In human trials, BDNF was quantified from serum [19,20,39,40,41,42]. Serum 25-hydroxyvitamin D [25(OH)D] levels were measured using the enzyme-linked immunosorbent assay (ELISA) method [20,39,40,42] or microparticle chemiluminescent immunoassay (CLIA) with the LIAISON^®^ system [19]. One trial did not include direct measurement of vitamin D levels [41]. One study combined vitamin D3 with magnesium (250 mg/day) [40], another combined vitamin D3 with zinc gluconate (30 mg/day) [39], and one study combined vitamin D3 with omega-3 fatty acids (1 g EPA and DHA/day), while the last did not assess vitamin D status [41].

Animal studies were included if they involved vitamin D supplementation and BDNF quantification in brain tissue. Depressive or cognitive-decline-like behavior in rodents was modeled using the chronic mild stress (CMS) or unpredictable chronic mild stress (UCMS) paradigms, middle cerebral artery occlusion (MCAO), post-stroke depression (PSD), scopolamine injections, or rat model of Lipopolysaccharide (LPS) induced an Alzheimer’s disease, which may have involved prolonged exposure to low-intensity, variable stressors over several weeks to induce anhedonia, behavioral despair, or cognitive decline. Behavioral outcomes, such as mood and cognitive performance, were assessed using specialized tests (SPT, sucrose preference test; FST, forced swim test; OFT, open field test; MWM, Morris water maze; PA, passive avoidance; T-maze test) (Table 3 and Table 4). In one study, functional brain effects were inferred from biochemical markers (e.g., oxidative stress, cholinergic activity) without conducting behavioral tests [29]. Serum concentrations of 25-hydroxyvitamin D3 [25(OH)D3] were measured using enzyme-linked immunosorbent assay (ELISA) [29,30,43,44,45]. Although some animal studies did not include direct measurements of serum or tissue vitamin D concentrations, specific supplemental doses of vitamin D or calcitriol were administered [46,47].

Exclusions included reviews, meta-analyses, in-vitro studies, multicomponent interventions lacking a vitamin D or BDNF component, conference abstracts without full text, as well as studies combining vitamin D with pharmacological treatments, or other interventions that could confound interpretation of vitamin D’s independent effects on BDNF except other microelements or clinical outcomes.

### 2.3. Search Strategy and Study Selection

PubMed, Cochrane CENTRAL, Web of Science, and Google Scholar were searched until 30 June 2025 with limits set to 2009–2025, English language, animals and humans. Initial yields were 867, 62, 475, and 570 records, respectively; snowballing added 10, giving 1984 records.

**Figure 1:**
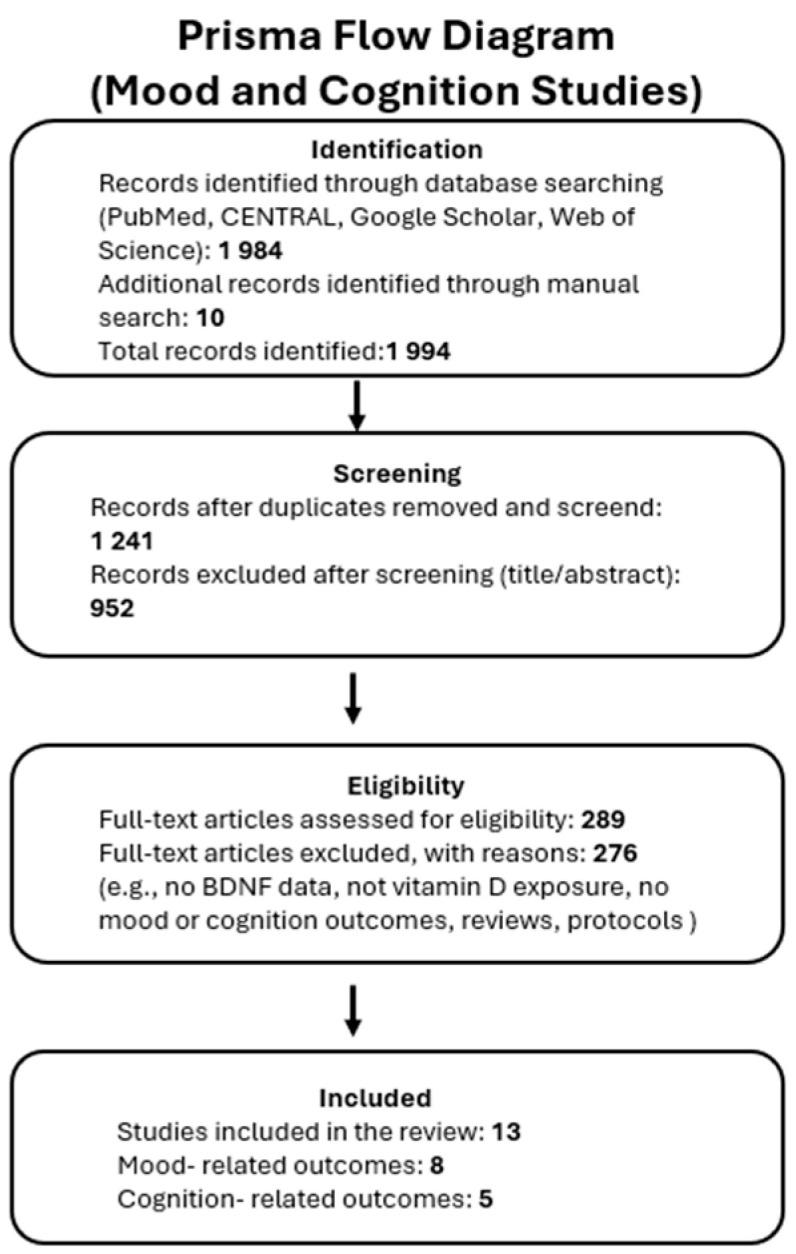
PRISMA flowchart diagram of study selection process.

After manual deduplication in Zotero, 1241 records underwent title/abstract screening, 289 full texts were assessed, and 13 studies met all criteria (Figure 1).

### 2.4. Data Extraction

The tables presented the study design, participant characteristics, vitamin D dosage/status, study methods, BDNF-related measures, mood and cognitive outcomes (including behavioral tests and biomarker assays), and key findings. Data were organized and formatted in Microsoft Word and then graphically digitized using Microsoft PowerPoint.

### 2.5. Quality Assessment and Data Synthesis

Randomized controlled trials (RCTs) were evaluated with the RoB 2 tool [48], observational studies were rated with the Newcastle–Ottawa Scale (NOS) [49], and animal studies used the SYRCLE risk of bias tool [50]. Overall, most included studies showed moderate risk of bias. Two reviewers (A.S.-R. and A.C.-W.) appraised each record independently, and disagreements were resolved by a third reviewer (K.M). Due to substantial heterogeneity across study designs, populations, and outcomes, meta-analysis was not performed. Findings are instead reported narratively and summarized in two tables (Vitamin D and Mood; Vitamin D and Cognition). The methodological quality of the review scored 26/30 for mood and 23/30 for cognition domains on the SANRA checklist (Table 1). A completed PRISMA 2020 checklist [33] is available in Table 2. Regarding study-level risk of bias, the three included RCTs [39,40,41] were rated as having “some concerns” of bias according to RoB 2, mainly due to insufficient detail in the selection of reported outcomes and incomplete description of the randomization process or blinding. Observational studies [19,20,42] received 8–9/9 stars on the NOS, mainly due to limited comparability between groups and incomplete outcome assessment reporting. All animal studies assessed with the SYRCLE tool [50] showed low risk of bias in domains related to baseline similarity and outcome completeness but unclear or high risk in domains such as sequence generation, allocation concealment, random housing, and blinding—primarily due to insufficient reporting. Given the small number of studies and the narrative synthesis approach, publication bias was not formally assessed, but its presence cannot be excluded.

## 3. Results

### 3.1. Effects of Vitamin D on BDNF and Mood

Eight studies met the eligibility criteria [30,39,40,41,42,45,46,47]. Three clinical trials [39,40,41] investigated the effects of vitamin D supplementation at doses of either 2000 IU/day or 50,000 IU/week. In two of these [39,40], baseline 25(OH)D concentrations were in the range of 16–39 ng/mL, falling below the threshold commonly considered sufficient. Baseline 25(OH)D levels were 16.33 ± 9.67 ng/mL in Abiri et al. and 26.07 ± 13.27 ng/mL in Yosaee et al. (2020), indicating insufficient vitamin D status in both samples [39]. Participants in Abiri et al.’s (2022) study demonstrated modest yet significant increases in circulating BDNF levels by 1.4% in the vitamin D-only group and 9.2% in the vitamin D + magnesium group, averaging approximately 7% across these intervention [40]. In the study by Yosaee et al. (2020), no significant changes in serum BDNF levels were observed following 12 weeks of supplementation with vitamin D alone or in combination with zinc [39]. Although both intervention arms showed numerical differences compared to baseline, these changes did not reach statistical significance, suggesting that vitamin D—either alone or co-administered with zinc—did not substantially affect circulating BDNF concentrations in this sample of obese individuals with depressive symptoms [39,40]. However, significant improvements in depressive symptoms were reported in both studies [39,40]. A study by Abiri et al. (2022) reported reductions of several (1.73 VitD and 1.84 VitD + Mg) points on the BDI-II scale compared to placebo [40]. According to the 3-day dietary recalls acquired throughout the intervention, no statistically significant difference was observed between the four groups in regards to dietary intakes of calories, macronutrients, and micronutrients, including magnesium and vitamin D [40]. In Yosaee et al. (2020) participants showed improved results of 3.87 (VitD) and 7.62 (VitD + Zn) points on the BDI-II scale [39]. In contrast, the study by Vyas et al. (2023) among older adults without clinical depression applied 2000 IU/day of vitamin D3 combined with omega-3 fatty acids [41]. Although BDNF levels were measured, no significant change was observed over 2 years, and percent changes were not reported. Participants were likely vitamin D-replete at baseline (>30 ng/mL) [41]. A reduction in incident depression was explored using the MINI and PHQ-9 tools, but no statistically significant association was found between vitamin D supplementation, BDNF levels, and depression prevention [41]. One cross-sectional study by a logistic regression analysis showed that a 1 ng/mL increase in serum vitamin D was significantly associated with a higher likelihood of having no depressive symptoms (PHQ-9 < 5) compared to having severe symptoms (PHQ-9 ≥ 15; OR = 1.076; 95% CI: 1.037–1.116; *p* < 0.001) [42]. This suggests that higher levels of 25(OH)D may offer a protective effect against depression. Additionally, a marginally significant interaction (*p* = 0.057) was observed between serum vitamin D and BDNF concentrations in relation to PHQ-9 scores, suggesting a potential synergistic effect [42]. Individuals with both higher 25(OH)D and BDNF levels exhibited the lowest depression severity, whereas those with lower levels of both biomarkers had the highest PHQ-9 scores [42]. It may point to a potential synergistic effect of vitamin D and BDNF—both involved in brain development—on depression risk [42]. One preclinical study demonstrated that vitamin D supplementation reduced depressive-like behaviors in rodent models of aging or chronic stress [47]. Yousefian et al. (2018) reported behavioral improvements—specifically, reversal of anhedonia in the sucrose preference test—without measurable changes in hippocampal BDNF, suggesting a BDNF-independent mechanism of action [47]. Similarly, Yang et al. (2024) found that vitamin D injections (400–1600 IU/week/mouse) significantly improved depression-like behaviors in adolescent mice subjected to Unpredictable Chronic Mild Stress (UCMS), despite no significant increase in hippocampal BDNF expression (*p* > 0.05) [30]. Notably, the antidepressant-like effects were only observed under stress conditions, indicating that vitamin D may interact with stress-responsive neurobiological pathways beyond BDNF, which warrants further investigation [30]. In contrast, Xu and Liang (2021) provided direct mechanistic evidence for a BDNF-mediated effect of active vitamin D. In a Post-stroke depression model (MCAO combined with UCMS), daily intracerebroventricular administration of calcitriol (25 μg/kg) over 4 weeks significantly increased hippocampal BDNF expression and reversed depression-like behaviors, including reduced sucrose preference and increased immobility in the forced swim test [46]. In this study, these antidepressant-like effects were abolished by co-administration of BDNF-binding protein (TrkB-IgG), indicating that the behavioral improvement was dependent on BDNF signaling in the hippocampus [46]. A similar effect was observed in a study by Koskhina et al. (2019) in menopausal CUMS rats, where reduced locomotor activity and a tendency to remain independent were found [45]. Furthermore, decreased BDNF concentrations and BDNF protein expression were observed in the hippocampus of these rats [45]. Vitamin D administered at a dose of 5.0 mg/kg reversed anhedonia- and depression-like states in the sucrose preference test (SPT)/forced swim test (FST) paradigms in menopausal rats with UCMS [45]. Moreover, the vitamin D application (5.0 mg/kg s.c.) restored the behavioral impairments observed in the open field test (OFT) [45]. In addition, biochemical assays found that vitamin D at this dose increased hippocampal BDNF and enhanced the hippocampal BDNF protein expression [45]. These data suggest that vitamin D at a dose of 5.0 mg/kg s.c. attenuated the UCMS-induced behavioral impairments, improved the hormonal state, as well as restored the serum vitamin D and neurotrophic factor levels in the hippocampus [45]. In contrast, vitamin D supplementation at a dose of 1.0 mg/kg exacerbated the behavioral disturbances, inducing more pronounced anhedonia-like and depression-like profiles, and significantly reduced BDNF concentrations, as well as the protein expressions of all neurotrophic factors in the hippocampus of the menopausal rats with UCMS [45] (Table 3).

**Table 3:** Studies assessing vitamin D, BDNF and mood outcomes.

No	Author (Year)	Design/Population	Vitamin D Exposure	Vitamin D Dose/Status/Baseline 25(OH)D ng/mL	BDNF Outcomes	Mood Assessments	Key Findings
1	Yosaee et al. (2020) [39]	RCT/Obese adults with mild/mod depression *n* = 140 >20 y	Vitamin D alone or with Zn	2000 IU/day for 12 weeks/26.07 ± 13.27 ng/mL	↔ in serum BDNF	↓ BDI-II	Vitamin D and Zn and vitamin D supplementation improved mood scale scores; no mediation tested.
2	Abiri et al. (2022) [40]	RCT/Obese women with mild/mod depression *n* = 102 20–45 y	Vitamin D alone or with Mg	50,000 IU/week for 8 weeks/16.33 ± 9.67 ng/mL	↑ in serum BDNF in vit D and Mg group	↓ BDI-II	Co-supplementation vitamin D and Mg improved mood scale scores and level of BDNF; no mediation tested.
3	Vyas et al. (2023) [41]	RCT/Older adults (late-life depression prevention) *n* = 400 ≥60 y	Vitamin D + omega-3 fatty acids	2000 IU/day for 2 years/baseline not reported	↔ in serum BDNF after 2 years	↔ MINI (DSM-IV) for incident MDD; PHQ-9 for symptoms	No effect on depression incidence or symptoms; BDNF did not change or mediate effect.
4	Goltz et al. (2018) [42]	Cross-sectional/Adults from general population (SHIP-TREND cohort) *n* = 3926 36–67 y	Vitamin D status	Mean 21.1 (14.4–29.9) ng/mL	↔ BDNF	↑ vitamin D–↓ PHQ-9	Higher level of vitamin D associated with lower depression severity.
5	Yousefian et al. (2018) [47]	Animal/CMS model in rats *n* = 42 ♂	Vitamin D i.p.	5 or 10 µg/kg, 2×/week for 5 weeks/baseline not reported	↔ BDNF in hippocampus (NS)	↑ Sucrose preference SPT	Vitamin D presented antidepressant-like effect—reversal of anhedonia; BDNF did not change or mediate effect.
6	Yang et al. (2024) [30]	Animal/UCMS adolescent model in mice *n* = 75 ♂	Vitamin D i.m.	400/800/1600 IU/week for 8 weeks/baseline not reported	↔ BDNF in hippocampus expression	↓ Immobility in FST; ↑ activity in OFT	Vitamin D prevented depression-like behavior; BDNF did not change or mediate effect.
7	Xu and Liang (2021) [46]	Animal study/PSD, MCAO + UCMS model in mice *n* = 32 ♂	Active vitamin D (calcitriol) i.c.v.	25 μg/kg/day for 4 weeks/baseline not reported	↑ Hippocampal BDNF expression (↑ protein and mRNA)	↑ SPT; ↓ immobility in FST	Vitamin D injection reversed depression-like behavior via ↑ hippocampal BDNF; blocked by TrkB-IgG ↓ anhedonia.
8	Koshkina et al. (2019) [45]	Animal study/UCMS and menopausal model in rats *n* = 49 ♀	Vitamin D s.c.	1.0, 2.5, 5.0 mg/kg/day × for 4 weeks/baseline 25(OH)D ≈ 15 µg L^−1^ (~15 ng mL^−1^)	5.0 mg/kg↑ hippocampal BDNF but 1.0 mg/kg ↓ hippocampal BDNF	5.0 mg/kg ↑ SPT, ↓ immobility in FST; various doses ↑ activity in OFT	High-dose of vitamin D normalized BDNF and fully reversed anhedonia- and depressive-like behavior, but low-dose worsened mood and ↓ BDNF, indicating dose-dependent role of hippocampal neurotrophins in vitamin D-linked mood regulation.

Abbreviations: 25(OH)D–25-hydroxyvitamin D; BDI-II, Beck Depression Inventory-II; BDNF, brain-derived neurotrophic factor; CMS, chronic mild stress; FST, forced swim test; i.c.v., intracerebroventricular injection; i.m., intramuscular injection; i.p., intraperitoneal; IU, international unit; Mg, magnesium; MDD, major depressive disorder; MCAO, middle cerebral artery occlusion; MINI (DSM-IV), Mini-International Neuropsychiatric Interview; OFT, open field test; PHQ-9, Patient Health Questionnaire-9; RCT, randomized controlled trial; PSD, post-stroke depression; s.c., subcutaneous; SHIP-TREND, Study of Health in Pomerania–Trent; SPT, sucrose preference test; TrkB-IgG, Tropomyosin receptor kinase B-immunoglobulin G fusion protein; UCMS, unpredictable chronic mild stress; vitD, vitamin D; y, year; Zn, zinc; ↑, increase; ↓, decrease; ↔, no significant change/association; ♂, males; ♀, females.

### 3.2. Effects of Vitamin D on BDNF and Cognitive Function

Five studies met criteria of examining the relationship between vitamin D and brain-derived neurotrophic factor (BDNF) in the context of cognitive outcomes [19,20,29,43,44]: one cross-sectional among older adults [19], one observational with child participation [20], two with vitamin D [29,43], and one calcitriol supplementation on animal models [44]. Quialheiro et al. (2023) in their cross-sectional study found that higher serum levels of vitamin D were significantly associated with better cognitive performance and increasing serum BDNF levels among older adults [19]. For every 10 ng/mL increase in serum 25(OH)D, MMSE scores increased by 0.6 points (adjusted Coef = 0.04; 95% CI: 0.001–0.007; *p* = 0.040) [19]. This corresponds to a ~2.4% improvement in cognitive function [19]. The authors also observed that higher levels of vitamin D were positively associated with serum BDNF concentration [19]. In that case, each 10 ng/mL increase in vitamin D was associated with an increase of approximately 230.9 pg/mL in serum BDNF, reflecting a ~14.8% increase in BDNF when comparing individuals with deficient and normal vitamin D status [19]. However, no significant association was found between serum BDNF and MMSE scores (*p* = 0.798) and the indirect effect of vitamin D on cognitive performance through BDNF (*p* = 0.917) [19]. However, higher levels of MMSE and BDNF in participants with higher vitamin D levels suggest a possible relationship through adjustment of the supplementation dose [19]. Next, a study by Dewi et al. (2025) showed that children (<2 years) with lower serum vitamin D levels (mean: 27.65 ng/mL) demonstrated significantly poorer performance in the ASQ-3 problem-solving domain (*p* = 0.005) [20]. No significant association was found between serum BDNF levels and cognitive outcomes, nor between vitamin D and BDNF concentrations (both *p* > 0.05), although mean BDNF levels were numerically lower in vitamin D-deficient children (≤32.7 ng/mL), suggesting a possible trend yet not reaching statistical significance [20]. An animal study by Khairy and Attia (2021) observed elevated hippocampal brain-derived neurotrophic factor (BDNF) following 5 weeks of vitamin D supplementation (500 IU/kg/day) in aging rats, along with favorable changes in oxidative stress and aging markers [29]. Specifically, vitamin D increased cognition markers, such as glutathione (GSH) levels, reduced malondialdehyde (MDA) and tumor necrosis factor alpha (TNF-α) concentrations, and decreased acetylcholinesterase (AChE) activity [29]. No behavioral or cognitive testing was conducted in this study [29]. A study on scopolamine-induced cognitive deficits in animals showed that scopolamine injection decreased BDNF concentrations in hippocampal tissue [43]. Furthermore, pretreatment with vitamin D doses of 1000 and 10,000 IU/kg increased BDNF levels, but the lowest dose of vitamin D was not effective [43]. Analyses also showed that scopolamine injection significantly impaired rats’ performance in the Morris water maze (MWM) and passive avoidance (PA) tests [43]. Again, vitamin D administration at doses of 1000 and 10,000 IU/kg improved cognitive performance in the MWM and PA tests [43]. Medhat et al. (2020) showed similar results; BDNF showed a significant decrease in the Alzheimer group compared to the control group, while there was an increase in their levels in the vitamin D group and significant increase in the combined vitamin D and exercise treated group compared to the Alzheimer group [44]. In the case of cognition, there was a significant increase in time consumed in the T-maze test in the Alzheimer group compared to the control group [44]. On the other hand, there was a significant decrease in the vitamin D group and combined vitamin D and exercise treated groups compared to the Alzheimer group, with the best results seen in the combined vitamin D and exercise treated group [44] (Table 4).

**Table 4:** Studies evaluating vitamin D, BDNF and cognitive outcomes.

No	Author (Year)	Design/Population	Vitamin D Exposure	Vitamin D Dose/Status/Baseline 25(OH)D ng/mL	BDNF Outcomes	Cognition Assessments	Key Findings
1	Quialheiro et al. (2023) [19]	Cross-sectional/older adults *n* = 576 ≥ 60 y	Vitamin D status	Mean ~26.5 ng/mL; categorized: <20, 21–29, ≥30 ng/mL	↑ vitamin D–↑ in serum BDNF	↑ vitamin D–↑ MMSE	Higher level of vitamin D associated with higher BDNF and better cognitive performance; BDNF not mediator.
2	Dewi et al. (2025) [20]	Cross-sectional/children *n* = 85 <2 y	Vitamin D status	Mean 27.65 ng/mL (10.5–39.8 ng/mL); cutoff ≤ 32.7 vs. >32.7 ng/mL	↑ vitamin D → ↑ serum BDNF	↑ vitamin D → ↑ gross motor, social, problem solving (ASQ-3)	Higher level of vitamin D associated with higher BDNF and better cognitive development; BDNF not mediator.
3	Khairy and Attia (2021) [29]	Animal study/Rats *n* = 60 ♂	Vitamin D oral supplementation	500 IU/kg/day for 5 weeks/baseline not reported	↑ BNFS in brain	Biochemical markers (BDNF, AChE, oxidative stress, caspase-3)	Vitamin D showed neuroprotective effects and improved biochemical markers of aging.
4	Mansouri et al. (2021) [43]	Animal study/scopolamine-induced cognitive deficit *n* = 50 ♂	Vitamin D i.p. (with scopolamine)	100, 1000, and 10,000 IU/kg for 3 weeks/baseline not reported	↑ BNFS in hippocampus	↑MWM, ↑PA	Vitamin D improved cognitive outcomes and BDNF levels; no mediation tested.
5	Medhat et al. (2019) [44]	Animal study/LPS-induced AD-like rats *n* = 50 ♀	Active vitamin D (calcitriol) i.p. and calcitriol and exercise	1 μg per kg of body weight/2× day for 4 weeks	↑ BDNF in brain	T-maze: ↓ time, ↑ % alternation	Vitamin D and vitamin D and exercise improved cognitive outcomes and BDNF levels; no mediation tested.

Abbreviations: 25(OH)D, 25-hydroxyvitamin D; AChE, acetylcholinesterase; ASQ-3, Ages and Stages Questionnaire, Third Edition; BDNF, brain-derived neurotrophic factor; i.p., intraperitoneal; LPA, lipopolysaccharide; MMSE, mini-mental state examination; MWM, Morris water maze; IU, international unit; PA, passive avoidance; UCMS, unpredictable chronic mild stress; y, year; ↑, increase; ↓, decrease; ↔, no significant change/association; →, association; ♂, males; ♀, females.

## 4. Discussion

This structured narrative review synthesized thirteen eligible studies (three human RCTs, three observational cohorts, and seven animal experiments) that collectively quantified vitamin D (supplementation or status), brain-derived neurotrophic factor (BDNF) levels, and mood and cognitive outcomes between 2009 and 2025. In RCTs with baseline 25-hydroxyvitamin D [25(OH)D] < 30 ng/mL, vitamin D supplementation ≥ 2000 IU/daily or 50,000 IU/weekly for ≥12/8 weeks increased circulating BDNF by ≈ +7% and reduced Beck Depression Inventory II (BDI-II) scores by 1.7–7.6 points [39,40]. However, two years of vitamin D supplementation (with omega-3 fatty acids) did not improve depressive symptoms [41]. Furthermore, modeling studies have confirmed the effect of vitamin D supplementation on mood but were inconsistent regarding the effect on BDNF levels. Animal studies correlated dose-dependent normalization of hippocampal BDNF and reversal of stress-induced anhedonia [45,46]. In the context of cognitive performance, observational studies have shown that a 10 ng/mL higher 25(OH)D concentration is associated with a 0.6-point improvement in the mini-mental state examination (MMSE) score and a 15% increase in serum BDNF concentration [19]. However, the tests did not show an association between BDNF and MMSE [19]. It has also been noted that in children under 2 years of age, low vitamin D levels may indicate certain cognitive problems [20]. In this case, animal studies confirmed a dose-dependent increase and normalization of BDNF concentration in the hippocampus and improvement of cognitive performance, as well as improvement of biochemical markers of aging [29,43,44].

Vitamin D traverses the blood–brain barrier and binds the nuclear vitamin D receptor (VDR), highly expressed in hippocampus, pre-frontal cortex, and limbic nuclei [6]. Liganded VDR heterodimerizes with retinoid-X-receptor, recruits co-activators, and enhances phosphorylation of the cyclic-AMP-response-element binding protein (CREB). The p-CREB complex up-regulates transcription of BDNF, increasing neuronal brain-derived neurotrophic factor (BDNF) [27]. BDNF acts as the canonical synaptic “tag” that converts early-phase long-term potentiation into its protein-synthesis-dependent late phase and consolidates declarative memory; blocking TrkB or silencing BDNF abolishes spatial- and fear-memory formation [9]. Vitamin D supplementation, rather than exogenous BDNF, prevents age-related long-term potentiation (LTP) decline in rats [26], underscoring its upstream, modulatory role. Additional pre-clinical work confirms this VDR-CREB-BDNF cascade; vitamin D elevates hippocampal BDNF in Alzheimer-model mice [28], reverses oxidative-stress-related BDNF loss in aging rats [29], and is summarized mechanistically by Bathina and Das (2015) [8]. Yet, an adolescent-stress model showed antidepressant-like behavior after vitamin D injections without parallel BDNF up-regulation [30], implying alternative anti-inflammatory or glutamatergic pathways may operate in certain contexts. Overall, vitamin D sits upstream of a mechanistic bottleneck centered on CREB-dependent BDNF transcription, integrating synaptic plasticity with emotion regulation. A review of 103 meta-analyses classified both low 25(OH)D and reduced peripheral BDNF levels as possible risk factors for depression, but downgraded the quality of evidence to low due to cross-sectional heterogeneity [51]. Conversely, a meta-analysis on antidepressant treatment arms reported a large peripheral-BDNF rise with clinical response [52]. Together with our structured review, these syntheses outline a plausible chain of association—vitamin D → BDNF → mood/cognition—while highlighting the need for longitudinal mediation analysis. Two studies show that supplementing with at least 2000 IU daily for several weeks can improve mood and increase serum BDNF levels [39,40]. Two RCTs—one conducted in obese women with mild to moderate depressive symptoms [40] and the other conducted in adults with obesity and mild depression [39]—have already shown that ≥2000 IU cholecalciferol daily for 8–12 weeks increases serum BDNF levels (≈ +7%) and reduces BDI-II by 2–8 points. The mood results are confirmed with higher levels of evidence: a dose–response meta-analysis including 31 randomized study arms showed that each 1000 IU/day vitamin D3 supplementation slightly reduced depressive symptoms in individuals with and without depression (SMD: −0.32, 95% CI −0.43 to −0.22; GEADE = moderate) [53]. Moreover, the greatest reduction occurred at higher dosage (8000 IU/day; SMD: −2.04, 95% CI −3.77 to −0.31). This study suggests that vitamin D3 supplementation may effectively reduce depressive symptoms in the short term, preferably between 8 and 24 weeks (SMD: −0.47, 95% CI −0.70 to −0.24; *n* = 15) [53]. Next, a comprehensive umbrella meta-analysis review summarizing 15 meta-analyses, which included 65 RCTs, and 31 observational (cohort and cross-sectional) studies showed that vitamin D supplementation in studies using doses > 5000 IU/day and intervention duration ≤ 20 weeks showed a stronger effect in reducing depressive symptoms [54]. Furthermore, the inverse association between lower serum vitamin D levels and depression was stronger among participants aged ≤ 50 years [54]. Furthermore, it is possible that individuals who have low serum 25(OH)D levels are expected to show greater benefits of vitamin D supplementation on depressive symptoms [54]. Longitudinal studies have indicated that low vitamin D levels were associated with developing depression in the future [54]. That was confirmed in the analysis that covered 25 trials with a total of 7534 participants and revealed that vitamin D had an effect on patients with major depressive disorder and on subjects with serum 25(OH)D levels ≤ 50 nmol/L. The pooled data from trials of vitamin D supplementation lasting ≥8 weeks and dosage ≤ 4000 IU/day indicated that vitamin D had an effect [55]. Smaller studies using lower doses or adjunctive medications point in the same direction; for example, a dose of 1500 IU daily as an adjunct to fluoxetine accelerated symptom remission in major depression [56], and a weekly dose of 50,000 IU improved PHQ-9 scores in women with type-2 diabetes [57]. However, the literature is not uniformly positive. In the present review, a large preventive study conducted among community-dwelling adults aged ≥60 years found that a dose of 2000 IU/day for two years, combined with omega-3 fatty acids, did not reduce the incidence of depression or alter BDNF [41]. Similarly, a network meta-analysis of 18 RCTs assessed that the mixed evidence suggests that vitamin D supplements have moderate effectiveness in alleviating depressive symptoms. The authors observed no moderating effects of vitamin D supplement duration and dose, serum 25-hydroxyvitamin D concentration at baseline, or changes in serum 25-hydroxyvitamin D concentration in the vitamin D group. However, in this 8-week study, patients with more severe depression responded better than those with less severe depression (*p* = 0.053) [58]. Factors such as baseline heterogeneity, co-nutrient, supplement or medical supplementation, gender, and season in the studies likely explain these discrepancies—a hypothesis confirmed in other studies [25,53].

The present review included observational studies in humans over 60 years of age [19] and in animal models of cognitive impairment [29,43,44], which confirmed the association between supplementation status or dose and the presence of cognitive impairment. Even studies in children show that vitamin D levels may be a factor associated with the development of cognitive impairment [20]. Available data on cognitive function show a similar pattern but are generally weaker. A 24-week Turkish study in older adults showed that vitamin D replacement may not improve cognitive function in older adults, even if vitamin D levels were raised to appropriate levels and adjusted for the severity of the deficiency, indicating the need for longer therapy to improve cognitive function [59]. A small RCT (*n* = 26) showed that 10 weeks of vitamin D supplementation resulted in increased cortical excitability, but these values were not different from placebo [60]. In the pooled studies, one observational study showed that each 10 ng/mL increase in serum 25(OH)D concentration was associated with a 0.6-point increase in MMSE in Brazilian seniors [19], and a Mendelian randomization analysis suggested a protective association between 25(OH)D levels and cognitive parameters [61]. Furthermore, a meta-analysis showed that 25(OH)D levels were inversely associated with the risk of both dementia and Alzheimer’s disease. The researchers observed a linear dose–response relationship, indicating that a 10 nmol/L increase in 25(OH)D levels could lead to a 5% decrease in the risk of dementia and a 7% decrease in the risk of Alzheimer’s disease [23]. A meta-analysis of five studies noted that the risk of dementia was associated with deficient serum vitamin D levels [62]. On the other hand, a review of 16 studies showed that vitamin D supplementation can improve cognitive outcomes in patients with mild cognitive impairment, but there is no evidence that it can prevent dementia or modify the course of Alzheimer’s disease [63]. Furthermore, an analysis of 24 studies found that there is insufficient evidence to suggest that vitamin D supplementation can improve cognitive function in people with Alzheimer’s disease [64]. The effect of vitamin D alone may be significantly greater in women than in men in terms of cognitive impairment [65]. In summary, the literature on mood now contains numerous converging RCTs and dose–response meta-analyses that support vitamin D supplementation, often with a threshold of ≥2000 IU—particularly in the setting of vitamin D deficiency or co-supplementation with magnesium and zinc. The field of these associations for cognitive health still awaits longer, adequately powered studies using objective domain-level outcomes. However, these available results suggest that the vitamin D-induced effect appears sufficient to alter cognitive symptoms within a few weeks, particularly in individuals struggling with severe vitamin D deficiency and cognitive problems. However, there is no convincing evidence for the prevention of dementia or Alzheimer’s disease.

In this review, a few studies on mood and cognition found an association between vitamin D supplementation or concentration and higher serum or hippocampal BDNF levels [19,20,29,40,43,44,45,46]. Some studies did not confirm this association [30,39,41,42,47], and one study in an animal model of menopause and stress showed that low-dose vitamin D supplementation may have the opposite effect [45]. The review indicates some potential benefits, such as confirming that BDNF incretion attenuates depression-like behavior and reverses anhedonia [46] or indicating a dose-dependent role of hippocampal neurotrophins in vitamin D-linked mood regulation [45]. However, most studies did not investigate or confirm the role of BDNF. The role of BDNF as a modulator of vitamin D supplementation on mood and cognitive measures is therefore unclear. Other studies have also noted that without neurotrophic readings, the mechanistic implications of changes in mood or cognition remain unclear. Studies have already shown that vitamin D-induced improvements are consistent with increases in BDNF levels when the biomarker is actually measured [9,27], and meta-analytic studies support BDNF as a potential marker of response [52]. However, conclusive clinical data on the mechanistic nature of BDNF that would explain its role as a modulator are lacking. Future studies should aim to assess—via serum or exosome—at least a clinical baseline and endpoint, and ideally determine optimal time points for BDNF measurements and increase evidence for the use of BDNF as a predictive biomarker in the assessment of cognitive ability and depressive states, as such evidence is lacking.

Most studies show that baseline vitamin D levels are insufficient in both adults [19,39,40] and children [20]. This vitamin D deficiency affects over one-third of adults in Central Europe and is worse during months of low sunlight. Large population surveys leave little doubt that vitamin D deficiency is the norm rather than the exception in Central Europe. In the pan-European analysis by Cashman et al. (2016), which comprised more than fifty-five thousand serum samples collected between latitudes 35° and 69° N, the Central-European belt—countries like Poland, Czechia, Slovakia, Hungary, Germany, and Austria—emerged as one of the most affected regions. Averaged across the calendar year, 40.4% of adults had circulating 25-hydroxy-vitamin D below 20 ng/mL, and 1% fell under the clinical-deficiency threshold of 12 ng/mL [66]. European individuals had serum 25(OH)D concentrations < 12 ng/mL on average in the year, with 17.7% and 8.3% in those sampled during the extended winter (October–March) and summer (April–November) periods, respectively [66]. The prevalence of vitamin D deficiency (i.e., 25(OH)D < 20 ng/mL) in U.S. adults is higher for women (35%) than for men (25%). Moreover, postmenopausal women with osteoporosis are especially likely to exhibit deficiency [67]. An analysis of 217 obese adolescents in the U.S.A. revealed that 55% of the patients were vitamin D deficient (defined as 25(OH)D levels < 20 ng/mL), while 22% had levels below 10 ng/mL. The prevalence of vitamin D deficiency (i.e., 25(OH)D < 20 ng/mL) in American adults is higher in women (35%) than in men (25%). Furthermore, postmenopausal women with osteoporosis are particularly at risk for deficiency [67]. Another study found that in 1606 men over the age of 65, 26% were deficient [25(OH)D < 20 ng/mL], and 72% were deficient (<30 ng/mL) [68]. So, targeting this group maximizes both public health significance and statistical power [66,69]. The consensus statement of Płudowski et al. (2013) lists strata in which deficiency is virtually guaranteed: unsupplemented infants, pregnant or lactating women, obese adults (BMI ≥ 30 kg/m), institutionalized elders, dark-skinned minorities at northern latitudes, and patients with malabsorption or chronic kidney or liver disease. In these cohorts the prevalence of 25(OH)D < 20 ng/mL ranges from 60% to >80%, and in unsupplemented infants it exceeds 90% by six months of age [69]. Therefore, future studies should intentionally enrich their samples with participants whose baseline 25-hydroxyvitamin D concentration is below 12 ng/mL [69,70]. Additionally, season or photoperiod must be controlled within study design and statistical analysis. At latitudes above 50° N, dermal synthesis of cholecalciferol ceases from October through March; ignoring this factor risks misclassifying both exposure and outcomes [66,69].

Gender—and in women, menopausal status—appears to be a consistent biological enhancer of the vitamin D → BDNF → mood/cognition cascade. A small, three-month pilot study of 20 postmenopausal women taking 8000 IU of calcifediol orally showed measurable cognitive benefits but decreased BDNF levels [71]. Synergy of exercise during estrogen withdrawal may be helpful. Aerobic training plus vitamin D3 (10,000 IU/kg/week) eliminated memory deficits in ovariectomized rats, whereas either intervention in isolation was insufficient [72]. The authors noted a parallel restoration of BDNF and CREB phosphorylation in the hippocampus, which reinforces the mechanistic overlap of estrogen and vitamin D signaling. Observational data on aging women also support this finding. In 72 Polish postmenopausal participants, higher levels of BDNF and Geriatric Depression Scale (GDS) were observed, as were the clock test results in current and overweight participants [73]. In another study, the authors suggest that physical activity levels may also influence certain biochemical markers and cognitive functions in postmenopausal women [74]. While the direct link between vitamin D and BDNF in menopause is not fully established, research suggests that estrogen’s influence on vitamin D activation and BDNF production could be relevant. Estrogen deficiency during menopause may lead to decreased BDNF levels, potentially impacting cognitive function and mood [75]. A review by Kalueff and Tuohimaa (2007) documents one possibility: that estradiol enhances VDR-dependent transcription. These findings suggest that declining estrogen levels unmask vitamin D sensitivity: when the primary estrogen-BDNF pathway is lost, vitamin D supplementation may partially compensate, restoring neurotrophin levels and improving mood or memory [7]. The clinical implications are clear: future RCTs should be stratified by menopausal stage or specifically targeted at peri- and postmenopausal women, a demographic in which the prevalence of depression and vitamin D deficiency is high, and pharmacological options are often limited.

In three studies with positive results on mood [39,40,41], and in one also on BDNF [40], where the largest decreases in BDI-II were observed, particularly significant relationships were seen with concomitant magnesium or zinc supplementation [39,40]. Similarly, in the case of cognitive impairment, an animal model with calcitriol and physical exercise demonstrated a stronger effect on brain BDNF levels and improved cognitive performance in tests in the group combining supplementation with exercise [44]. A meta-analysis of antidepressants showed a greater increase in BDNF in women [52,76], which is consistent with estradiol-induced VDR transactivation [7]. Fluoxetine may also enhance VEGF, BDNF, and cognition in patients with vascular cognitive impairment and dementia [77]. In ovariectomized rats, only high-dose calcitriol normalized BDNF and behavior [45]; vitamin D combined with aerobic training restored spatial memory [72]. And in an animal model of Alzheimer’s disease, cognitive performance scores were statistically significantly improved in the supplementation and exercise group [44]. Results of other reviews have shown that supplementation with vitamin D, probiotics (especially Lactobacillus species), and PUFAs would most likely reduce cognitive decline and dementia [78]. Magnesium, zinc, omega-3, and B vitamins enhanced the neurotrophic and clinical benefits induced by vitamin D in other studies as well [79]. And multicomponent training in individuals with mild cognitive impairment (MCI)/dementia provided a cognitive SMD ≈ 0.4 with a proportional increase in BDNF [80]. Probiotics increase BDNF levels, especially when combined with vitamin D-fortified dairy [81,82]. Supplementation and physical training multiply the vitamin D → BDNF → clinical response axis, generating greater and faster benefits than vitamin D alone. Therefore, future studies should consider co-supplementation of vitamin D with cofactors such as magnesium, zinc, B vitamins, probiotics, and long-chain omega-3 fatty acids, and with structured physical exercise, to achieve greater and faster effects on mood and cognitive health, but also in increasing BDNF.

Mood-related apathy or low drive may impair traditional, effort-dependent cognitive batteries—a problem confirmed by several studies in this review. The Turkish study by Ates Bulut et al. (2019) relied on paper-based executive tests but did not report any tests of effort or motivational validity; because baseline Geriatric Depression Scale scores averaged 9 ± 3 points, subthreshold depression may impair actual cognitive development [59]. Similarly, the Brazilian cross-sectional analysis by Quialheiro et al. (2023) used the MMSE without adjustment for comorbid depressive symptoms, which were present in 22% of their sample [19]. In contrast, one of the included studies and Pirotta et al. (2015) minimized this threat by using objective computerized measures of transcranial magnetic stimulation or clinician-rated MINI interviews, which are less susceptible to poor task engagement [41,60]. These discrepancies suggest that future studies of vitamin D cognitive function should incorporate objective measures of effort accuracy (e.g., reliable digit span, memory malingering test) or move to computerized, time-stamped platforms that capture response latency and consistency. Adherence to supplementation recommendations and regimens, another equally significant source of error in cohorts with depression or cognitive impairment, was clearly quantified in only a few included studies. Abiri et al. (2022) and Yosaee et al. (2020) collected unused capsules at each visit and remeasured serum 25(OH)D concentrations—a dual approach that confirmed >90% adherence [39,40]. The two-year preventive study by Vyas et al. (2023) utilized monthly pill counts and annual 25(OH)D monitoring, documenting high levels of adherence [41]. However, in another Spanish cohort of adults with mild cognitive impairment or mild dementia, adherence to chronic medications—including vitamin D supplements, as defined by pill count—decreased by more than 10% at 12 months [83]. Participants reported forgetfulness, disorganization, and lack of understanding of instructions. This demonstrates that the same cognitive deficits we hope to alleviate can also hinder regular supplementation, leading researchers to underestimate any true biological benefits of vitamin D [84]. Such gaps hinder internal validity and may explain the variance in cognitive performance. Therefore, future studies should introduce objective adherence monitoring (electronic pill containers, e-diaries, monthly 25(OH)D retests) and caregiver-assisted dosing, retain participants in intention-to-treat (ITT) analyses, and combine this with statistical adjustment for baseline and treatment variables so that non-adherence is treated as outcome-relevant information rather than as missing data.

Diet quality places vitamin D within a broader “proneurotrophic” nutrient matrix, rather than as a standalone factor. NHANES 2013–2015 data on 1344 Americans aged >6 years show that higher dietary vitamin D intake is associated with improved cognitive performance, particularly in tests related to animal fluency and memory, as well as lower levels of depression in elderly individuals [85]. Similar results regarding the inverse association with depressive symptoms for vitamin D intake, especially from fatty fish, were obtained in a 3-year observational study of 81,189 women aged 50–79 years at baseline. Furthermore, cross-sectional analyses based on baseline data showed that women with the highest intakes of vitamin D from food sources had a significantly lower prevalence of depressive symptoms, as assessed by the Burnam scale, compared to women who reported intakes of <100 IU vitamin D/day [86]. Similar results were observed for children in the presented review; in children under 2 years of age, higher serum vitamin D concentrations were associated with better cognitive development, especially motor and problem-solving skills, as well as with higher BDNF levels [20]. A diet based on appropriate whole foods reinforces these observations regarding dietary patterns. A review of several recent reviews identified multiple observational studies (both cross-sectional and longitudinal) and intervention trials that provide consistent and converging evidence for the positive impact of the Mediterranean diet (MeDi), the Dietary Approaches to Stop Hypertension (DASH) diet, and the Mediterranean-DASH Intervention for Neurodegenerative Delay (MIND) diet on brain health and cognition. However, benefits have also been demonstrated for the ketogenic diet, intermittent fasting, and weight management diets [87]. Among 960 older Chicago citizens followed for over 4.5 years, a strict one-serving daily serving of leafy green vegetables and foods rich in phylloquinone, lutein, nitrate, folate, α-tocopherol, and kaempferol, which are part of the Mediterranean, DASH, and MIND diets, was linearly associated with slower cognitive decline. The rate of decline in those consuming 1–2 servings daily was equivalent to being 11 years younger compared to those who rarely or never consumed leafy green vegetables [88]. On the other hand, ultra-processed, nutrient-poor diets are associated with lower BDNF levels and a higher risk of depression. This association is thought to be mediated by factors such as gut health, inflammation, and the effects of processed food components on brain function [89]. These findings indicate that vitamin D exerts its strongest mood- and cognitive-supporting effects in a plant-based diet rich in fish that also provides omega-3 fatty acids, magnesium, and polyphenols—cofactors known to synergize with the vitamin D–BDNF pathway. Future intervention studies should therefore quantify baseline diet quality, control for macronutrient and antioxidant intake, and test factorial models combining vitamin D supplementation with the Mediterranean or MIND diet and structured physical exercise.

Host genetics offer a further explanation for the heterogeneity observed in vitamin D studies. Common variants of the vitamin D receptor—FokI, BsmI, and TaqI—shape emotion and cognition in late life [90], while the BDNF Val66Met polymorphism alters neurotrophin secretion and susceptibility to mental illness [91]. Moreover, Mendelian randomized studies further support a causal role of vitamin D pathways in cognition: genetically higher 25(OH)D concentration was associated with a lower risk of Alzheimer’s disease [61]. Therefore, future studies should genotype, among others, VDR and Val66Met at baseline and treat their variants as distinct effect modifiers rather than merely confounders.

The structured narrative review has its strengths: it relies on a multi-database search strategy—PubMed, CENTRAL, Web of Science, and Google Scholar—that identified nearly 2000 unique records and minimized selection bias. Adherence to PRISMA-2020 reporting standards, risk-bias assessment, and high SANRA methodological scores (26/30 for mood; 23/30 for cognition) further enhance clarity. A second strength is the emphasis on mechanism: only quantitative studies on vitamin D and brain-derived neurotrophic factor (BDNF) were retained, allowing the review to link clinical changes to plausible biological pathways. Third, the review includes both human clinical trials and preclinical models (three RCTs, three observational cohorts, seven animal experiments), providing a translational perspective lacking in purely clinical syntheses. Finally, mood and cognitive function were treated as distinct clinical problems, yet reflecting the true comorbidity of diseases and the role of vitamin D and BDNF as a modulator in their pathogenesis, allowing for comprehensive conclusions to be drawn for adult populations.

However, the evidence base has significant limitations. Only thirteen studies met all eligibility criteria, and only three were randomized, placebo-controlled trials—all single-center and lasting no longer than twelve weeks [39,40,41]. Such a small sample size increases imprecision and precludes quantitative meta-analysis. Another limitation is methodological heterogeneity; BDNF was measured in serum or brain tissue using different ELISA platforms (e.g., Abiri et al., 2022 [40] vs. Quialheiro et al., 2023 [19]), and most cognitive outcomes were obtained using subjective screening tools (MMSE, ASQ-3) rather than specific objective tools that minimize human error. Reliance on peripheral markers creates additional uncertainty, as serum BDNF and 25(OH)D concentrations may not perfectly reflect hippocampal concentrations. Assessment of risk of bias revealed moderate concerns; studies did not always provide complete information on randomization or selective reporting, and several animal studies did not describe allocation concealment. The review included only thirteen geographically limited studies, which limits the generalizability of the results or their adjustment to geographical latitudes. Moreover, interpretation of BDNF changes should be cautious, as its variability due to study type (plasma vs. serum), circadian rhythm, and confounding factors such as physical activity or systemic inflammation, combined with uncertainty regarding its role as a causal mediator versus a correlative biomarker [92], may limit mechanistic conclusions. Finally, few studies adjusted for seasonality or genotype, and not all included adherence, which may confound results in trials with depression or cognitive impairment.

Future studies should focus on recruiting participants with serum 25(OH)D concentrations below 30 ng/mL and analyze outcomes by gender, menopausal stage, and key polymorphisms in the VDR and BDNF. Furthermore, the dose–response curve should be further refined. Comparative studies comparing physiological doses (800–2000 IU daily^−1^) with pharmacological regimens (≥50,000 IU weekly^−1^) should monitor both total and free 25(OH)D concentrations, as well as 1.25(OH)_2_D, to precisely define the window of exposure that optimizes neurotrophic growth. The role of BDNF as a mediator should also be clearly tested. Repeated or even serial measurements of serum BDNF or exosomal BDNF concentrations—at rest and after completing tasks in individual tests and at different time points during the intervention—will reveal whether BDNF actually transmits a vitamin D signal that improves mood or cognitive function. Outcome measures should also be refined. Combining depression or cognitive impairment scales with computerized cognitive batteries and hippocampal MRI imaging will yield domain-specific and structural endpoints that are less susceptible to bias. Interventional co-supplementation studies, e.g., vitamin D with magnesium, B vitamins, omega-3, probiotics, exercise, or specific dietary patterns, should also be considered, and microbiome sequencing should be integrated to identify synergistic biological pathways. It is essential to consider seasonality in future studies to determine whether endogenous synthesis is present or not confounding the intervention effects. The final element should consider adherence levels and methods for monitoring adherence to intervention regimens. In this case, implementing caregiver monitoring may be most beneficial to ensure that dosing accuracy no longer limits the observed benefits of vitamin D use. From a geroscience perspective, both vitamin D synthesis and BDNF expression decline with age, in parallel with processes such as neuroinflammation, cerebrovascular aging, mitochondrial dysfunction, and impaired neurogenesis [93]. These changes contribute to the pathophysiology of late-life depression and cognitive decline, aligning with broader concepts in geroscience and pathology that emphasize the interconnectedness of systemic inflammation, vascular pathology, and neurodegeneration in aging [94]. Beyond its neurotrophic actions, vitamin D also supports endothelial function, regulates cerebral blood flow, and maintains blood–brain barrier integrity [95]. Cerebrovascular dysfunction can produce mood and cognitive symptoms overlapping with those attributed to low BDNF [92,96], suggesting that vascular and neurotrophic pathways may act independently or synergistically in modulating clinical outcomes.

In summary, this synthesis provides a clear, mechanism-oriented overview of the vitamin D–BDNF–mood/cognition axis, but its conclusions are limited by the limited and methodologically diverse literature, highlighting the need for larger, randomized controlled trials (RCTs) stratified by season and genotype that utilize objective adherence tracking and domain-level cognitive endpoints.

## 5. Conclusions

1. **Vitamin D may increase BDNF.** High-dose protocols (≥2000 IU daily or 50,000 IU weekly) increase circulating or hippocampal BDNF levels by 7% in deficient humans and stressed rodents.
2. The **clinical effects of vitamin D** supplementation and its concentration on BDNF **are more pronounced** in their effects **on mood than on cognition impairment**. Each increase in BDNF levels corresponds to a decrease of several points on depression scales, while cognitive improvement is smaller and occurs only after longer-term supplementation or combined supplementation.
3. **Targeted correction to levels of 30–40 ng/mL is a cost-effective and effective strategy.** Vitamin D deficiency affects nearly half of women, the elderly, and those living above 49° north latitude. Achieving healthy vitamin D levels in these groups will offer the greatest public health benefits.
4. **Co-supplementation may prove crucial.** Combining vitamin D with magnesium, zinc, omega-3 fatty acids, probiotics, or structured exercise doubles the neurotrophic and symptomatic response.
5. **Future research must consider important determinants.** Seasonality, the role of genotype, gender, and objective monitoring of adherence should be considered in future projects, along with repeated BDNF testing and the use of objective methods and tools to provide definitive evidence of the effectiveness of vitamin D in neuropsychiatric therapy.

## Figures and Tables

**Table 1:** SANRA quality scores.

SANRA Criterion (0–5)	Mood Section	Cognition Section
1. Clearly defined aim	5	5
2. Search strategy	4	4
3. Presentation of studies	4	3
4. Critical appraisal	4	3
5. Interpretation/conclusions	4	4
6. Relevance/significance	5	4
Total (max 30)	26	23

**Table 2:** PRISMA 2020 checklist.

Section/Item	Description	Completed
Title	Identify the report as a review	Yes
Abstract	Structured summary (PRISMA-Abstract)	Yes
Rationale	Describe rationale	Yes
Objectives	Provide explicit statement of objectives	Yes
Eligibility criteria	Specify study characteristics	Yes
Information sources	All databases, date of last search	Yes
Search strategy	Full search strings	Yes
Selection process	Methods and independent reviewers	Yes
Data collection process	Methods of extraction	Yes
Risk of bias	Specify tools used	Yes
Synthesis methods	Methods of synthesis	Yes
Reporting bias assessment	Assess risk of reporting bias	Partial
Certainty assessment	Certainty of evidence (GRADE)	NA
Results—Study selection	Flow diagram	Yes
Results—Study characteristics	Tables	Yes
Results—Risk of bias	Presentation of risk of bias	Yes
Results—Synthesis	Narrative synthesis	Yes
Discussion	Interpretation, limitations	Yes
Other info—Registration	Registration and protocol	No (narrative)
Other info—Funding	Sources of support	Yes

## Data Availability

Data are available in the articles cited in the References section.

## Abbreviations

The following abbreviations are used in this manuscript:

25(OH)D	25-hydroxycholecalciferol (25-hydroxyvitamin D)
AD	Alzheimer’s disease
ASQ-3	Ages and Stages Questionnaire, Third Edition
BDI-II	Beck Depression Inventory, Second Edition
BDNF	Brain-derived neurotrophic factor
BMI	Body mass index
CI	Confidence interval
CREB	cAMP (cyclic adenosine monophosphate) response element-binding protein
DALYs	Disability-adjusted life years
DASH	Dietary Approaches to Stop Hypertension
DNA	Deoxyribonucleic acid
ELISA	Enzyme-linked immunosorbent assay
GDS	Geriatric Depression Scale
IU	International unit
LTP	Long-term potentiation
MCI	Mild cognitive impairment
MeDi	Mediterranean diet
MIND	Mediterranean-DASH Intervention for Neurodegenerative Delay
MMSE	Mini-mental state examination
MRI	Magnetic resonance imaging
NHANES	National Health and Nutrition Examination Survey
NOS	Newcastle–Ottawa Scale
PHQ-9	Patient Health Questionnaire-9
PRISMA	Preferred Reporting Items for Systematic Reviews and Meta-Analyses
PUFA	Polyunsaturated fatty acids
RCT	Randomized controlled trial
RoB 2	Risk of Bias 2
SANRA	Scale for the Assessment of Narrative Review Articles
SMD	Standardized mean difference
SYRCLE	Systematic Review Centre for Laboratory animal Experimentation
TrkB	Tropomyosin receptor kinase B
VDR	Vitamin D receptor
WHO	World Health Organization
